# Screening and Brief Intervention for Unhealthy Drug Use: Little or No Efficacy

**DOI:** 10.3389/fpsyt.2014.00121

**Published:** 2014-09-02

**Authors:** Richard Saitz

**Affiliations:** ^1^Department of Community Health Sciences, Boston University School of Public Health, Boston, MA, USA; ^2^Clinical Addiction Research and Education (CARE) Unit, Section of General Internal Medicine, Department of Medicine, Boston Medical Center, Boston University School of Medicine, Boston, MA, USA

**Keywords:** screening and brief intervention, unhealthy drug use, illicit drug, efficacy, randomized trials, counseling, identification, primary care

## Abstract

Unhealthy drug use ranges from use that risks health harms through severe drug use disorders. This narrative review addresses whether screening and brief intervention (SBI), efficacious for risky alcohol use, has efficacy for reducing other drug use and consequences. Brief intervention among those seeking help shows some promise. Screening tools have been validated though most are neither brief nor simple enough for use in general health settings. Several randomized trials have tested the efficacy of brief intervention for unhealthy drug use identified by screening in general health settings (i.e., in people not seeking help for their drug use). Substantial evidence now suggests that efficacy is limited or non-existent. Reasons likely include a range of actual and perceived severity (or lack of severity), concomitant unhealthy alcohol use and comorbid mental health conditions, and the wide range of types of unhealthy drug use (e.g., from marijuana, to prescription drugs, to heroin). Although brief intervention may have some efficacy for unhealthy drug users seeking help, the model of SBI that has effects in primary care settings on risky alcohol use may not be efficacious for other drug use.

## Introduction

Screening and brief intervention (SBI) for unhealthy alcohol use in primary care is among the most effective and cost-effective of preventive services (1). Unhealthy alcohol use is the target and is defined as the spectrum from use that increases the risk for health consequences through a diagnosable alcohol use disorder (2). No randomized trials have compared SBI to no SBI for alcohol. Numerous studies in primary care find efficacy for BI vs. no BI among patients identified by screening for modest reductions in self-reported alcohol consumption (3). Although efficacy has yet to be demonstrated for moderate to severe alcohol use disorders (also known as dependence) (4–6), the conceptual model includes referral to specialty treatment as one of the goals of brief intervention for those with more severe conditions.

Brief counseling has also been applied to other health behaviors, such as medication adherence, nutrition, tobacco use, and physical activity with some success (7). Since the principles may be the same regardless of the health behavior there has been optimism that SBI will have efficacy for other drugs. Unhealthy drug use is defined as use of illicit drugs or potentially addictive medications more than prescribed or without a prescription. The US government has spent approximately half a billion dollars in the past decade on clinical programs to screen and provide brief intervention for alcohol and other drugs (8). Given the impact of drug use on health and the need to address drug use in general health settings, knowing whether SBI has efficacy for preventing or reducing drug use and consequences takes on great importance.

Observational studies have suggested possible effectiveness of drug SBI. For example, Madras et al. (9) conducted a before/after study and 6 months after screening found a 68% decrease in self-reported drug use and improvements in overall health, employment, criminal justice involvement, and housing status. The effect size is much larger than any ever seen in a randomized trial of a similar intervention and is not plausibly attributable to SBI. A well-done observational study with matched controls found that exposure to SBI in the emergency department was associated with subsequent linkage to specialty addiction treatment (10). However, one must interpret these studies with great caution and not take them as evidence for efficacy because there are many explanations for decreased use besides SBI, such as regression to the mean, assessment reactivity, secular trends and natural history, self-change, and others.

## Efficacy of Brief Intervention for Drug Use

Randomized trials of brief intervention in people seeking help can suggest possible efficacy, but they should not be taken as evidence for SBI in unselected patients identified by screening. In a meta-analysis that included interventions some consider longer than brief (e.g., 1–2 h, often multi-session), drug BI had an effect size of 0.29 in studies of motivational interviewing (11). In studies of treatment-seeking people, motivational interventions have often had efficacy (12–19), though at least one high-quality study found no efficacy (20). Several studies have found benefit in a focused area – reducing prescriptions for benzodiazepines (21–23). BI studies in special populations have had mixed results (24). A computerized intervention and voucher decreased use of drugs, except marijuana, in postpartum women (25). A single feedback session decreased drug (though not alcohol or marijuana) use in homeless adolescents (26). Another study at youth agencies found no effect on ecstasy or cocaine use (27). But other studies have found some effects in youth in mandated treatment and high schools (28–30).

## Efficacy of SBI for Drug Use in General Health Settings

Most people who use drugs and/or have drug use disorders neither seek nor receive treatment (31). As a result, interventions with the greatest potential to affect health in this area must have efficacy in general health settings, especially in primary and preventive care settings. Patients who receive these interventions should include those identified by screening, not only those who seek help for these conditions specifically. The US Preventive Services Task Force, a leading agency that rates and recommends preventive services based on the best evidence in the literature, reflects this view (32).

Few studies have tested the efficacy of SBI in general health settings compared with no-intervention control groups, and results are largely disappointing (see Table 1). Key issues are that to draw valid conclusions, adequate follow-up rates are needed, biological testing should confirm self-reports, and outcomes beyond use are of importance. These issues are also relevant to alcohol SBI and although numerous studies with self-report outcome in primary care for unhealthy alcohol use have confirmed efficacy, few have found effects on biological or clinical outcomes, raising questions about whether the evidence base for alcohol SBI is sufficient to suggest that efficacy for alcohol or other substances. One study of drug SBI compared computer to live human brief intervention for drugs in primary care and included biological outcomes. Results were similar in both groups, with some outcomes favoring the computer group, but drug use did not change much in either group [three points decrease in global alcohol, smoking, and substance involvement screening test (ASSIST) score], and with no control group, efficacy could not be determined (33).

**Table 1:** **Randomized trial evidence regarding drug screening and brief intervention in adult general health settings^a^ that include at least some primary care patients**.

Citation	Intervention	Result (between group differences at follow-up)	Comment
Gelberg et al. (34)	Very brief advice, video doctor, and two booster sessions	Less frequent (4 days) drug use at 3 months; effect larger among more severe	78% Follow-up; attention control; no biological testing; excluded those with likely moderate to severe disorder
Roy-Byrne et al. (35, 48)	Single BI with 1 week phone booster done by social workers	3, 6, 9, and 12 months outcomes. No significant differences in days drug use or drug use severity	Biological testing; 87% follow-up
Saitz et al. (36, 37)	Single 10–15 min health promotion advocate/health educator BI 45-min psychologist BI with one booster	6-month outcomes. No differences in days drug use or drug use severity, health-related quality of life, emergency department or hospital utilization or HIV risk behaviors	Biological testing; 98% follow-up
Humeniuk et al. (38)	Single BI largely done by clinic staff (some by researchers in Brazil)	Seven points or smaller difference in drug use risk scale with 338 points theoretical maximum at most sites except US where control group had greater decrease in the score	86% Follow-up; no biological testing; excluded those likely to have moderate to severe disorder^b^
Bernstein et al. (39)	Single BI done by health promotion advocate	5% Absolute risk increase in cocaine abstinence; 9% risk increase in opioid abstinence	Biological testing; 82% follow-up^b^

*^a^Two additional studies have been done exclusively in emergency department settings. One had 58% loss to follow-up and found no benefit of SBI (40). The other, a multi-site trial, has not yet had results published (41)*.

*^b^Some participants in primary care (see text for details)*.

Bernstein et al. (39) reported results of a randomized trial among adults with cocaine or heroin use identified by screening, in women’s health, homeless, and urgent care clinics. Most (82%) had follow-up though one in five were excluded because they had no evidence of drug use by hair testing at study entry. Opioid abstinence was 9% greater and cocaine abstinence 5% greater in the brief intervention groups though there was no increase in receipt of addiction treatment.

In a small randomized trial (*n* = 59) among adolescents in primary care in Brazil, BI reduced marijuana and ecstasy related problems (42). In project CHAT (*n* = 42), teenagers with drug use consequences who had brief intervention reported less marijuana use than controls (43). In three randomized controlled trials among adolescents in the emergency department, BI decreased recidivism related to drug consequences, increased abstinence, or increased entry into treatment (44–46). One randomized trial in adults using psychoactive prescription drugs in a general hospital found two counseling sessions were associated with decreased drug use though whether the use was misuse or appropriate use for pain was not clear (47).

Perhaps the most relevant study to the question at hand is the World Health Organization randomized trial in five countries in 731 adults reporting risky drug use (excluding those with more severe use). Patients were recruited from sexually transmitted disease clinics, dental and walk-in clinics, and community medical care sites. Very small differences were found favoring the BI group on a global scale of drug use risk of uncertain clinical importance and results at the US site were negative (point estimates favored the control group) (38). More specifically, both groups began at a global ASSIST score of 36; the BI group reduced to 30 while the control group reduced to 32, a 2-point difference in a scale with a maximum score of 338. In the US, the global score decreased by nine points in the control group and five points in the BI group (*p* = 0.11, *n* = 218). In India, decreases were 4 vs. 8 points, respectively (*p* = < 0.005, *n* = 177); Brazil 2 vs. 7 (*p* < 0.005, *n* = 165); and in Australia 0 vs. 8 (*p* < 0.001, *n* = 170).

Several recent studies provide information on the efficacy of SBI for drugs. Saitz et al. tested the efficacy of SBI for drugs randomizing 528 primary care patients identified by screening to one of two brief motivational interventions delivered by trained health educators or psychologists, the latter of which included a booster session (36, 37). At 6 months with 98% follow-up, there were no significant differences in drug use outcomes overall or in analyses stratified by drug type or drug use severity. This study used hair testing to corroborate self-report. Gelberg et al. have preliminarily reported a randomized trial of drug SBI in primary care, with 78% 3-month follow-up (34) (registered at www.clinicaltrials.gov NCT01942876). Patients with more severe risky use were excluded. The intervention was <5 min of brief advice, then a video doctor, and two follow-up counseling sessions. Results were a greater reduction in drug use days (by four) in the intervention vs. the control group, particularly among those who used drugs more frequently. Validity concerns include the absence of laboratory testing to corroborate outcomes, which leaves social desirability bias as a likely explanation for the results (given the largely negative findings in trials with biological outcomes and large changes in drug use in observational studies). In addition, the intervention was not particularly brief as it included several repeat contacts (and those with two or more such contacts had better outcomes).

Another large study in primary care also in the US has been published (35, 48), and outcomes were verified by laboratory testing. Roy-Byrne et al. (48) randomized 868 adults identified by screening to a single brief motivational intervention and a 10-min booster at 2 weeks by phone. At follow-up (≥87% at 3, 6, and 12 months), there were no significant differences in frequency of use or in drug use severity.

Although not in primary care, a large randomized trial of SBI in emergency department patients found no differences in drug use outcomes, though 58% of participants were lost to follow-up, substantially limiting the ability to draw firm conclusions from the results (40). A large multi-site study of SBI in emergency departments in the US with a minimal screening control group and a no-intervention control group is also underway (41). Both of these emergency department studies used biological testing to corroborate self-report outcomes.

## Conclusion and Implications

There is little evidence that SBI for drugs other than alcohol and tobacco will have efficacy in adult primary care settings. Three trials have been done exclusively in primary care. One with 98% follow-up of a large sample and biologically corroborated outcomes is entirely negative. Another with 87% follow-up and biologically corroborated outcomes also found no effects. Another trial, smaller and with short-term and substantially lower follow-up, has positive findings but no biological outcome confirmation. A large multi-site emergency department study is as yet unpublished. A single site study was negative and substantially limited methodologically. A study in mixed settings including urgent care did find small reductions in heroin and cocaine use corroborated by biological outcomes. A hospital study of prescription drug use was difficult to interpret. The WHO multi-site study found results of questionable clinical importance that were inconsistent across country (and negative in the US). In general, these results do not support the hypothesis that SBI has efficacy for drug use.

This narrative review may have some limitations. It is a narrative review based on searching Google scholar for randomized trials of drug SBI, attendance at national and international meetings where such research is likely to be presented, review of studies funded by the National Institute on Drug Abuse (nihreporter.gov) and search of the clinical trials.gov registry, and by review of a current systematic review that has full methodology published (49). While not a full systematic review, it is very unlikely that an important clinical trial of drug use SBI has been missed though that is a possibility.

If health behaviors are similar, why might SBI not have efficacy for drug use? Drug use may well be different from other health behaviors and from risky alcohol use specifically. First of all, drug use is often illegal and socially proscribed. As a result, when it is addressed in a health setting, the patient is using drugs despite this social sanction, whereas for a number of other health behaviors that respond to brief interventions, the patient’s behavior may be normative; when they realize their personal risks they decide to make changes. Most people who use drugs are aware of some risks. Drug use may be more severe than some other health behaviors. Drug use could range from occasional marijuana use (perceived by patients as safe) to prescription opioid misuse (a very complex problem that often involves chronic difficult to treat pain), to injection heroin or cocaine use. It seems unlikely that single brief counseling sessions could adequately address this range, even if the goal is to link patients to further and more specialized treatment.

Future research should always include biological outcomes. New approaches might address multiple risk behaviors and involve prioritizing them for intervention. Such approaches might then focus on subgroups of patients, such as those with prescription drug misuse or marijuana use. New approaches will also very likely need to test multi-contact longitudinal interventions of the type known to be more efficacious for alcohol.

For clinicians, the absence of efficacy of drug SBI does *not* mean that identifying and addressing drug use in patients is not important. It simply means that doing so by screening using validated tools to detect unhealthy use may not immediately lead to reduced drug use and problems after a brief intervention. Patients with symptoms need to be asked about drug use just as they would be asked about medication use, use of complementary therapies (e.g., herbal treatments), and dietary habits. Such information is critical both to appropriate diagnosis of medical and psychiatric conditions and to safe prescribing, particularly of psychoactive and addictive medications.

The evidence for efficacy of drug SBI is lacking. Editorialists, leaders at the US National Institute on Drug Abuse and the National Institute on Alcohol Abuse and Alcoholism, the largest supporters of substance use research in the world, say it is time to “go back to the drawing board” regarding screening and BI for drugs in primary care (50). Clinicians need to address drug use but cannot rely on SBI, a seemingly simple solution, to solve what is, in fact, a complex problem. Researchers need to find more effective means in general health settings to address what is a common preventable cause of death in the world.

## Conflict of Interest Statement

The author declares that the research was conducted in the absence of any commercial or financial relationships that could be construed as a potential conflict of interest.
